# The effects of an unsupervised water exercise program on low back pain and sick leave among healthy pregnant women – A randomised controlled trial

**DOI:** 10.1371/journal.pone.0182114

**Published:** 2017-09-06

**Authors:** Mette G. Backhausen, Ann Tabor, Hanne Albert, Susanne Rosthøj, Peter Damm, Hanne K. Hegaard

**Affiliations:** 1 Department of Obstetrics, Copenhagen University Hospital (Rigshospitalet), Copenhagen, Denmark; 2 Department of Gynecology and Obstetrics, Zealand University Hospital, Roskilde, Denmark; 3 Research Unit Women`s and Children`s Health, The Juliane Marie Centre, Copenhagen University Hospital (Rigshospitalet), Copenhagen, Denmark; 4 The Institute of Clinical Medicine, Faculty of Health and Medical Sciences, University of Copenhagen, Copenhagen, Denmark; 5 The Modic Clinic, Odense, Denmark; 6 Section of Biostatistics, Department of Public Health, University of Copenhagen, Copenhagen, Denmark; Stanford University School of Medicine, UNITED STATES

## Abstract

**Background:**

Low back pain is highly prevalent among pregnant women, but evidence of an effective treatment are still lacking. Supervised exercise–either land or water based–has shown benefits for low back pain, but no trial has investigated the evidence of an unsupervised water exercise program on low back pain. We aimed to assess the effect of an unsupervised water exercise program on low back pain intensity and days spent on sick leave among healthy pregnant women.

**Methods:**

In this randomised, controlled, parallel-group trial, 516 healthy pregnant women were randomly assigned to either unsupervised water exercise twice a week for a period of 12 weeks or standard prenatal care. Healthy pregnant women aged 18 years or older, with a single fetus and between 16–17 gestational weeks were eligible. The primary outcome was low back pain intensity measured by the Low Back Pain Rating scale at 32 weeks. The secondary outcomes were self-reported days spent on sick leave, disability due to low back pain (Roland Morris Disability Questionnaire) and self-rated general health (EQ-5D and EQ-VAS).

**Results:**

Low back pain intensity was significantly lower in the water exercise group, with a score of 2.01 (95% CI 1.75–2.26) vs. 2.38 in the control group (95% CI 2.12–2.64) (mean difference = 0.38, 95% CI 0.02–0.74 p = 0.04). No difference was found in the number of days spent on sick leave (median 4 vs. 4, p = 0.83), disability due to low back pain nor self-rated general health. There was a trend towards more women in the water exercise group reporting no low back pain at 32 weeks (21% vs. 14%, p = 0.07).

**Conclusions:**

Unsupervised water exercise results in a statistically significant lower intensity of low back pain in healthy pregnant women, but the result was most likely not clinically significant. It did not affect the number of days on sick leave, disability due to low back pain nor self-rated health.

**Trial registration:**

ClinicalTrials.gov NCT02354430

## Introduction

Low back pain is a frequent condition in pregnant women with a prevalence varying from 50 to 90% worldwide [1,2]. Both prevalence and pain intensity increase during pregnancy [3,4] and low back pain impacts negatively on the ability to perform daily activities [3]. Women with low back pain are more likely to take sick leave during pregnancy [5]. It is therefore important to focus on how to reduce low back pain in pregnant women.

The etiology of low back pain is still not clear, but it is in general perceived as multifactorial [6]. Studies show that increased mobility of the pelvic girdle is present in pregnant women, which causes instability and may be one of the factors of low back pain [7]. Supervised stabilizing exercises have shown to have a positive effect on pain intensity in pregnant women with pelvic girdle pain [8,9]. Randomised controlled trials (RCT) investigating the effect of supervised physical exercises on low back pain have shown conflicting evidence in preventing low back pain [10–12] but have shown to decrease low back pain intensity [13–15] and sick leave [10,12,14,15]. However a recent systematic review concluded that several studies have methodological problems and that high quality studies are still lacking [16]. To our knowledge, no study has previously investigated the effect of unsupervised exercise.

Pregnant women are recommended to be physically active 30 minutes a day [17] and observational studies suggest that physical exercise before and during pregnancy is associated with a lower risk of low back pain [18,19], but pregnant women tend to reduce their level or change type of physical exercise during pregnancy and some discontinue physical exercises [20,21]. This underlines the importance of the choice of activity type when planning exercise interventions for pregnant women. Activities such as swimming and exercises in water increase among pregnant women [22] and are considered comfortable and safe to do [23,24]]. In 2011 The Danish Rheumatism Association developed an exercise program, called AquaMama, for healthy pregnant women to use in public swimming pools nationwide. It aims to strengthen the larger muscle groups (legs, abdominal, back, hips, buttocks, arms and shoulders). No attempts have previously been made to investigate the effect of this exercise program. A feasibility study with 30 pregnant participants tested the AquaMama program and showed positive results in regard to recruitment, compliance with the program and the pregnant women´s experience of an unsupervised water exercise program [23]].

Our hypothesis was that strengthening exercises performed in water from 20–32 weeks of gestation have a positive effect on low back pain and sick leave in pregnant women. This study aimed to assess the effect of an unsupervised water exercise program on low back pain intensity and days on sick leave among healthy pregnant women in the setting of a RCT.

## Materials and methods

### Study design and participants

This trial was a randomised, controlled, parallel-group trial. It was performed at the Department of Obstetrics, University Hospital Rigshospitalet, Copenhagen, Denmark. Recruitment took place in the period from October 2013 until May 2015 and participants were followed until August 2015. The hospital serves as a primary birth facility for women of Copenhagen city and is a tertiary referral center with around 5,900 deliveries annually. Pregnant women were eligible for participation if they were healthy, 18 years or older, Danish speaking and between 16–17 gestational weeks. The exclusion criteria were: women with multiple pregnancies, prepregnancy body-mass index (BMI) > 29 kg/m^2^, medical or obstetrical complications, conditions contraindicating physical activity, women who had been diagnosed with pelvic girdle syndrome (either in the current or a previous pregnancy) and women with substance abuse problems. Pelvic girdle syndrome occurs in approx. 5% of all pregnant women and the risk of recurrence if diagnosed in the first pregnancy is high. It is defined as daily pain in all three pelvic joints confirmed by objective findings [25]. Individual supervised exercise is recommended for women with this condition [6].

The study was conducted in accordance with the Declaration of Helsinki. Written informed consent was obtained from all participants before enrolment. The trial was approved by the Scientific Committee of the capital region of Denmark (December 12, 2012 with journal nr: H-3-2012-132) and the Danish Data Protection Agency (2007-58-0015). The trial was registered at Clinical Trials.gov with the identifier: NCT02354430. As we were not aware of the importance of registration being completed before enrollment of the first participant, the study was first registered during participant enrolment. The authors confirm that all ongoing and related trials for this drug/intervention are registered.

#### Randomisation and masking

The participants were randomly assigned to either the water exercise or the control group (ratio 1:1) at baseline by a computer-generated random sequence, using random permuted block sizes (two, four or six) created by Public Health and Quality Improvement, Central Denmark Region, Aarhus. The Public Health and Quality Improvement had no other involvement in the project and concealed the sequence. The research midwife entered the participant’s social security number into the computer program (Trialpartner) on a laptop and this resulted in the participant was allocated to a group. As the randomization was performed by a secure computer program the allocation was thereby concealed until group assignment. Because the intervention consisted of exercise and as the investigators were engaged in the recruitment and inclusion of participants, neither participants nor investigators were blinded to group allocation. The water exercise group and the control group both received standard prenatal care, which in Denmark is free of charge and consists of; three visits at the general practitioner, five visits at a midwife and two ultrasound scans. Health promotion advice and guidance is given at these visits including advice on physical exercise according to the national guideline which is in accordance with international guidelines (Healthy pregnant women are advised to exercise at least 30 minutes of moderate intensity per day) [17]. The control group received two tickets for mother/baby-swimming when returning the follow-up questionnaire at 32 weeks of gestation.

#### Procedures

Among women with a normal nuchal translucency scan in 11–12 weeks (defined as: risk of Trisomy 21 < 1:300 and no malformations detected), 180–200 pregnant women were selected in blocks based on their due date each month in the study period and assessed for eligibility. Approximately 80 of these potential participants were invited by a posted letter, which included a detailed description of the trial. Inclusion procedure of each block of participants lasted about 2 to 3 weeks (block size varying between 22 and 35 women) and was timed in order to allow women allocated to water exercise to participate in an introductory session at approx. 20 weeks of gestation. The rest of the women were excluded according to the exclusion criteria. The procedure was performed once or twice a month for a total of 18 times, until the desired number of participants had been included.

After one week, the research midwife contacted the women by telephone or email to ensure that the invitation regarding the trial had been received and to answer any questions. Women interested in participating were invited to a face-to-face visit. At this visit, the women were given oral information about the trial and two provocation tests were performed in order to exclude women with pelvic girdle syndrome–the Trendelenburg test and the posterior pelvic pain provocation test [6]. If one of the tests was positive, the woman was referred to a physiotherapist and excluded if pelvic girdle syndrome was diagnosed. Subsequently, a self-administrated baseline questionnaire was filled out and the randomisation was performed immediately afterwards. The women who were randomised to the water exercise group received additional information concerning the time and place of the introductory session prior to commencement of the water exercise.

The water exercise intervention was performed between 20 and 32 gestational weeks and initiated by an introductory session at a public indoor swimming pool, followed by 12 weeks of unsupervised exercises twice a week. The introductory session consisted of a theoretical part led by two of the authors (MGB and HKH), where the participants were given theoretical counselling about general exercise recommendations during pregnancy and shown short movie clips of the six AquaMama water exercises. Subsequently, the women were given practical instructions by specially trained coaches while performing the exercises in water. The participants were encouraged to keep a training logbook that was handed out during the introductory session. A brush-up session was offered twice during the water exercise period to give an opportunity to ask questions regarding the exercises. Engagement in other physical activities were encouraged and allowed, due to national recommendations of 3.5 hours of weekly exercise [17]. Free access was given to seven public swimming pools in Copenhagen city. Short weekly motivating emails were sent to remind the participants to follow the water exercise program.

An exercise session consisted of: four swimming laps (100 m in total) as a warm up, followed by the six AquaMama exercises (described below) and finished with another four laps. The six exercises were performed in two series and required two foam dumbbells, a swim belt and a kickboard. Short movie clips of each exercise are available at: https://www.youtube.com/watch?v=F2uWMmtDD2w&list=PL10D1C9FDEF43F91F

**MamaSurf:** The feet were held stationary side by side on top of a kickboard. The exercise started from full hip and knee extension. The hips and knees were flexed and the ankles dorsiflexed allowing the kickboard to rise parallel to the surface of the water. After the hips were maximally flexed, the hips and knees were again extended and the ankles plantar flexed to push the kickboard further under the water until the hips and knees were fully extended again. This sequence was repeated 30 times.

**MamaPendul:** This exercise started in an upright position with arms stretched out with a 90-degree shoulder abduction and foam dumbbells in the hands. The participant flexed the hips and knees allowing the trunk to rotate in the water towards a supine position. Whilst rotating, the hips and knees were slowly extended until the participant was lying supine on the surface of the water. From this fully extended position, the hips and knees were flexed again and the trunk rotated back to upright and then further to a prone position where the knees and hips were extended again. This sequence of prone and supine extensions was repeated 20 times.

**MamaJogging:** The exercise was performed wearing a swim belt. The participant floated with the body as upright as possible and jogging movements were imitated in the lower extremities combined with reciprocal boxing movements of the arms. By performing these movements the body was propelled slowly forwards. The exercise was performed for two minutes and after a short break it was performed again, five times in all.

**MamaLift:** The handle of a single dumbbell was held horizontally on the surface of the pool. The arms were rotated such that the backs of the hands were facing upwards. The exercise started by pushing the dumbbell under water with extended elbows in the midline until it made contact with the body. The dumbbell was then slowly allowed to return to the surface. Afterwards, the dumbbell was pushed with stretched arms downwards and laterally to the left hip before it was allowed to rise slowly to the surface and finally downwards and laterally to the right hip. The sequence was repeated 12 times.

**MamaBoxing:** Standing position with the feet slightly apart and lightly flexed knees, so that the shoulders were at water level. The elbows were extended and shoulders flexed to 90 degrees with each pronated hand holding a dumbbell. The arms were flexed, one pulling the dumbbell towards the body, whilst the other was extended pushing the dumbbell directly away from the body and vice versa in a boxing pattern. This sequence was repeated 20 times.

**MamaBiceps:** The participant abducted her arms 90 degrees with extended elbows, and the dumbbells were held vertically in both hands with the palm of the hand facing forwards. Holding the dumbbells just below water level, the elbows were flexed until the dumbbells reached the chest and then extended again until the arms were once more out to the side. This sequence was repeated 20 times.

The exercises were performed at a moderate pace, corresponding to the Borg scale 11–13 and 14–15 [26]]. Each training session was estimated to last approximately 45 minutes. The participants were offered access to a closed Facebook group so they could share their experiences.

#### Outcomes

Data were obtained at baseline (18–20 weeks) by a self-administrated questionnaire and at follow-up (32 weeks). The primary outcome was the intensity of low back pain, measured at follow-up by the Low Back Rating Scale consisting of three numeric 11-point box scales (pain now, worst pain in the past two weeks, and average pain in the past two weeks), where 0 indicated no pain and 10 indicated worst pain imaginable. An average of the three measures constituted a score for each woman [27]]. According to the European guidelines low back pain is defined as pain between the 12^th^ rib and the gluteal fold and includes pelvic girdle pain [6]. A drawing of a woman marked with this definition of low back pain was shown in the questionnaire.

The secondary outcomes were self-reported days spent on sick leave, disability due to low back pain and self-rated general health. An open answer was provided to state the cause of sick leave, divided into three categories: pregnancy related, not pregnancy related or due to the work environment. Physical disability due to low back pain was measured by the 23-item Danish version of The Roland Morris Disability Questionnaire [28]] with yes/no answers, and the total score therefore scored from best to worst on a 0–23 point scale. The minimal important change is considered 5 points or a 30% reduction from baseline. Self-rated general health status was measured by EQ-D5 [29]]. It comprised five dimensions: mobility, self-care, usual activities, pain/discomfort and anxiety/depression. Each dimension had three levels: 1 = no problems, 2 = some problems, 3 = extreme problems; an index score was given from these answers. Finally, the EQ VAS was used to self-rate health on a vertical, visual analogue scale ranging from 0–100 mm, where the endpoints are labeled best imaginable health state (100) and worst imaginable health state (0). The history of low back pain was measured by a single question with a yes/no answer and how often it occurred (daily, weekly, monthly, occasionally or rarely). The use of pain medication within the past week was also stated. Compliance (the number of times the participant completed a session) was obtained by the questionnaire at follow-up and by logbooks. Data on sociodemographic characteristics, medical and obstetric history, smoking status and data regarding the present delivery were obtained from patient records. Engagement in exercise (yes/no), type of exercise and hours spent on each type weekly was obtained before the pregnancy, at baseline and at follow up. The items used were similar to the ones used in the Danish National Birth Cohort (1996–2002) [30] and in the Copenhagen pregnancy Cohort (2013-) [22], it is a modified version of the Minnesota Leisure-Time Physical Activity Questionnaire, which has been validated in non-pregnant women [31]. Exercise was defined as any physical activity that is planned, structured, and repetitive for conditioning any part of the body, but also included cycling used as means of transportation.

#### Statistical analysis

Sample size calculation was based on the following conditions: The highest pain score at baseline was 5.8 and SD = 3.0 [14,32] and a two-point reduction on a numerical pain scale of 0–10, was defined as clinically significant [33]. Furthermore, it was estimated that 50% of participants would follow the program for at least 75% of the sessions (high participation, 19–24 sessions) while 35% would follow 50–75% (moderate participation, 12–18 sessions) and 15% would follow less than half of the program (low participation) [14,34]]. We hypothesized that the effect of high participation in the intervention would lead to a reduction of 2 points, moderate participation would lead to a reduction of 1 point, while low participation would not result in a reduction. This gives an overall reduction in the intervention group of 1.35 points (0.50 * 2 + 0.35 * 1 + 0.15 * 0 = 1.35). To detect a difference in pain level of 1.35 (SD = 3.0) and with a power of 90%, a significance level of 5% (two-sided) the number of participants in each group is 105. As we expected 70% of the participants to experience low back pain, a dropout rate of 25% and 27% would not to answer the questionnaire at follow-up, the study was initially estimated to include 275 participants in each group. Inclusion of participants was stopped before 550 participants were included as the dropout rate was much lower than expected (<5%).

When preparing the manuscript, we realized that we had used the proportion of women experiencing low back erroneously. Only the 70% experiencing low back pain will benefit from the intervention resulting in an overall reduction in mean of 0.7*1.35 = 0.95 in the intervention group, thereby needing 211 participants in each group. Still, this higher number of participants was recruited to the study due to the low drop-out rate.

The outcomes were not normally distributed, however in order to report the effect of the intervention with confidence intervals and due to the large sample size, the parametric comparisons using the t-test were considered valid. To investigate the sensitivity towards the assumption of the outcome being normally distributed, the complete case analysis of the primary outcome was repeated using the non-parametric Kruskal-Wallis test as well as a permutation t-test (identical conclusions, results not shown). Per-protocol analysis was applied for women who completed the intervention (≥ 75% of the sessions, high participation). Due to missing data, and to perform intention-to-treat analyses, missing data techniques were applied for the analyses of the primary and secondary outcomes: Multiple imputation was used to create and analyze 50 multiple imputed data sets. Incomplete outcomes were imputed under fully conditional specification [35]]. In the imputation models, all outcomes as well as variables predictive of the primary outcome and/or the missingness mechanism were included (randomization, age, BMI, parity, education, baseline low back pain, exercise (prepregnancy) (yes/no), exercise at baseline (yes/no), history of low back pain (yes/no), self-rated general health (EQ-D5) at baseline as well as exercise at follow-up). Extreme right skewed outcomes were log(x+1)-transformed (days spent on sick leave). Quantitative variables were imputed using predictive mean matching, binary variables using logistic regression and ordinal variables using proportional odds logistic regression models. Based on the imputed data sets, the mean difference in outcomes between the two groups were determined including confidence intervals and p-values based on Rubin’s rule. Analyses were performed using SPSS IBM version 22, R version 3.2.0, and imputations were generated and analyzed using the mice 2.25 package. P-values less than 0.05 were considered significant.

## Results

**Fig 1:**
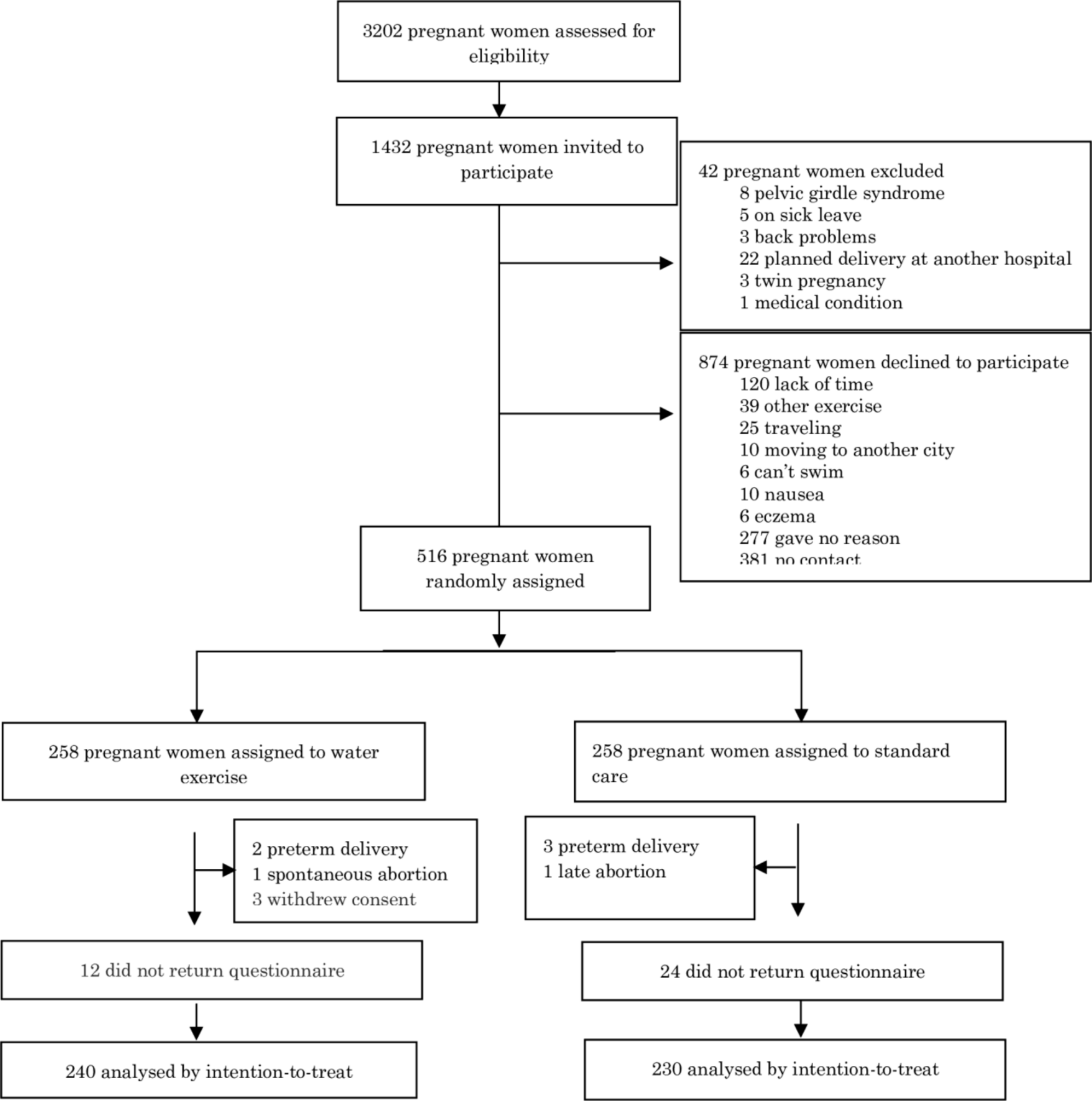
Participant flowchart.

In the period from October 2013 until May 2015, we screened 3202 pregnant women for eligibility (Fig 1). A total of 1770 pregnant women were excluded and 1432 were initially invited to participate. We randomly assigned 516 pregnant women to either unsupervised water exercise (n = 258) or control (standard prenatal care) (n = 258). Three women withdrew from the study: one found the exercises uncomfortable to perform, one preferred to do another kind of physical exercise and one had inflammation of the arm, which made execution of exercises impossible. Five women gave birth before follow-up at 32 weeks of gestation, one had a spontaneous abortion (21 weeks) and one had an induced abortion (22 weeks) due to a fetal malformation and were therefore excluded from follow-up. Outcomes were obtained for 91% of the participants at follow-up. A total of 46 (9%) participants were lost to follow-up (Fig 1). Participants lost to follow-up were less likely to be engaged in any physical exercise at baseline (relative risk = 2.7 95% CI 1.4–5.1 p = 0.005).

**Fig 2:**
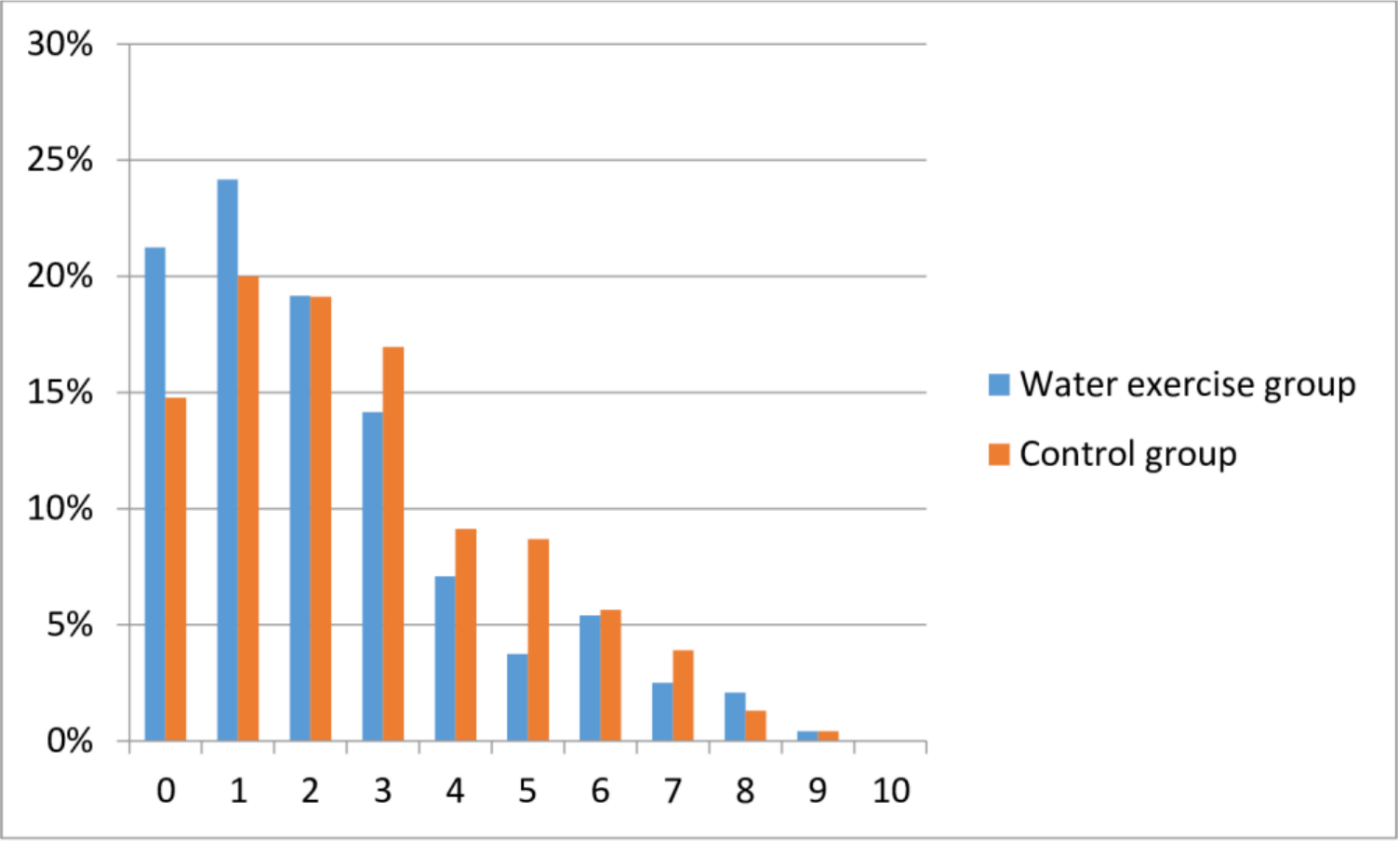
Histogram showing the distribution of primary outcome at follow-up. Low back pain intensity (numeric rating scale) and the distribution in both groups in percentages

The baseline characteristics of the study population showed no differences between the two groups, (Table 1). The mean age was 31 years (SD 4.2), the majority had a higher education (86%), were non-smokers (99.6%) and 83% were exercisers at baseline. We found a statistically significant effect of the water exercise on primary outcome (Table 2), but the result was not clinically significant. In the complete case analysis the low back pain intensity was 0.38 (95% CI 0.02–0.74 p = 0.04) lower in the water exercise group and an equivalent result was found when performing the analysis including multiple imputations. Per-protocol analysis of women who completed the intervention showed similar results -0.43 (95% CI -0.88–0.02 p = 0.06). More women reported no low back pain in the water exercise group at follow-up (21% vs. 14% p = 0.07) (Fig 2).

**Table 1:** Baseline characteristics of study participants by randomisation group.

N = 516			Water exercise	Control
	n		(n = 258)	(n = 258)
**Demographic**				
**Age (years)**	516	Mean (SD)	31.4 (4.3)	30.6 (4.1)
**Body mass index (kg/m²)**	516	Mean (SD)	23.8 (3.7)	23.5 (2.5)
**Nulliparous**	516	N (%)	190 (74)	185 (72)
**Co-habiting**	511	N (%)	236 (92)	240 (93)
**Years in school**	481			
**<12 years**		N (%)	22 (9)	16 (7)
**≥12 years**		N (%)	217 (91)	226 (93)
**Lifestyle factors**				
**Exercise (prepregnancy)**	513	N (%)	237 (93)	237 (92)
**Exercise baseline**	486	N (%)	212 (88)	216 (88)
**Smoking (at conception)**	510	N (%)	27 (11)	21 (8)
**Smoking (at10 weeks)**	511	N (%)	0	1 (0.4)
**Low back pain**				
**Low back pain intensity**	513	Median (min-max)	0.67 (0–7.33)	0.67 (0–8)
**History of low back pain**	513	N (%)	72 (28)	77 (30)

Low back pain is pain intensity measured by numeric rating scale. Exercise (prepregnancy) is exercise three month before conception defined as any physical activity that is planned, structured, and repetitive for conditioning any part of the body.

**Table 2:** Primary outcome.

N = 516					
		Water exercise	Control	Mean difference	p value
	n			(95% CI)	
**Primary outcome**					
**Low back pain intensity**	470	2.01 (1.75–2.26)	2.38 (2.12–2.64)	0.38 (0.02–0.74)	0.04
Analysis based on MI*					
**Low back pain intensity**	516	2.02 (1.77–2.27)	2.40 (2.15–2.66)	0.39 (0.03–0.74)	0.03

Low back pain intensity measured on three numeric rating scales in 32 weeks of gestation (follow up). Results are presented as means with 95% confidence intervals for complete case and multiply imputed data.

*Multiple imputation.

We found no difference in the secondary outcomes between groups (Table 3). The median number of days on sick leave was four in both groups (p = 0.83) and 19 (8%) women in both groups spent days on sick leave due to low back pain. The analyses based on multiple imputation of missing outcomes gave identical conclusions (results not shown). The groups did not differ in maternal or infant outcomes (Table 4) except for mode of delivery where planned Caesarean section, occurred more often in the water exercise group (9% vs. 2% in the control group). The most frequent indication for the planned Caesarean sections in the water exercise group was maternal request. One woman reported discomfort while performing the exercises and withdrew consent.

**Table 3:** Secondary outcomes.

			Water exercise	Control	p value
	n		(n = 258)	(n = 258)	
**Secondary outcomes**					
**Sick leave, yes**	466	N (%)	103 (39.9)	115 (44.6)	0.84
Sick leave (days)*	453	Median (min-max)	4 (1–72)	4 (1–180)	0.95
**Disability (RMDQ)**	454	Mean (95% CI)	3.14 (2.55–3.72)	3.44 (2.84–4.04)	0.47
**General health (EQ-5D)**	463	Mean (95% CI)	0.83 (0.81–0.84)	0.82 (0.80–0.83)	0.36
**GH-VAS**	465	Mean (95% CI)	82.8 (81.0–84.6)	80.8 (79.0–82.6)	0.12

Analysis was performed by the intention to treat principle and shown as complete case.

*The Kruskal-Wallis test was used for sick leave (days) and represent days on sick leave during the study period.

RMDQ: Roland Morris Disability Questionnaire. EQ-5D: EuroQol questionnaire on general health and GH-VAS: visual analog scale for self-rated general health.

**Table 4:** Mode of delivery, maternal and infant outcomes.

	n		Water exercise	Control	p value
**Maternal**					
**Gestational age (days)**	496	Mean (SD)	280.3 (12.3)	281.3 (12.6)	0.64
**Preeclampsia**	499	N (%)	7 (2.7)	8 (3.1)	0.57
**Gestational diabetes mellitus**	499	N (%)	1 (0.4)	1 (0.4)	-
**Preterm delivery**	496	N (%)	6 (2.5)	10 (4.0)	0.34
**Mode of delivery**					
**Vaginal delivery**	500	N (%)	201 (81)	224 (88)	
**Caesarean section (planned)**	500	N (%)	21 (9)	4 (2)	
**Caesarean section (emergency)**	500	N (%)	25 (10)	25 (10)	0.002
**Infant**					
**Birth weight (g)**	480	Mean (SD)	3549 (531)	3540 (531)	0.66
**Lenght (cm)**	478	Mean (SD)	52 (2.3)	51.5 (5.4)	0.14
**Apgar score (< 7 at 5 minutes)**	469	N (%)	3 (1.2)	0	0.12
**PH < 7.10**	456	N (%)	0	2 (0.8)	0.5

Chi-square was used to test the difference between groups.

The compliance with the water exercise was as follows: 98% (253) participated in the introductory session and 40% (104) participated in more than 75% of the exercise sessions, 34% (88) participated in more than half of the exercise sessions, 17% (43) participated in less than half of the exercise sessions, 7% (18) was lost to follow-up and an additional 2% (four women) did not respond to the question. At follow-up, women were equally physically active 89% vs. 85% (p = 0.18) and hours spent on exercise per week were also similar between groups (p = 0.57). Types of exercise were equally distributed between groups with exception of swimming, exercise in water, resistance training and other exercise. In all 17 women in the control group were engaged in water exercise at follow-up (Table 5). In analysis excluding women who were engaged in resistance training and in other* exercise no change in the overall result was found (mean difference was 0.41, 95% CI: 0.03–0.79, p = 0.03) vs. 0.38 (0.02–0.74, p = 0.04 for the whole material) (Table 2).

**Table 5:** Exercise, time spent on exercise and types of exercise at follow-up at 32 weeks.

	n		Water exercise	Control	p value
Exercisers^¤^	448	N (%)	209 (89)	182 (85)	0.18
Hours per week^#^	435	Mean (SD)	5.5 (4.4)	5.2 (4.0)	0.57
Types of exercise^¤^	448	N (%)			
**Cycling (transport)**			115 (50)	114 (54)	0.38
**Water exercise**			97 (42)	17 (8)	<0.001
**Swimming**			82 (35)	51 (24)	0.01
**Brisk walking**			82 (35)	93 (44)	0.06
**Yoga**			49 (20)	49 (23)	0.45
Other*			17 (7)	32 (15)	0.01
**Fitness**			10 (4)	7 (3)	0.58
**Resistance training**			9 (4)	20 (9)	0.02
**Running**			4 (2)	2 (1)	0.48
**Spinning**			3 (1)	2 (1)	0.73
**Horseback riding**			2 (1)	0	0.18

*dancing, gymnastic for pregnant women and pilates. Exercise was defined as any physical activity that is planned, structured, and repetitive for conditioning any part of the body.

^#^ T-test

^¤^ Chi-square

## Discussion

To our knowledge, this is the first randomised controlled trial to investigate the effect of an unsupervised water exercise intervention on low back pain among healthy pregnant women. We showed that an unsupervised water exercise program had a modest positive effect on the primary outcome (intensity of low back pain) in healthy pregnant women, but this was most likely not clinically significant. The water exercise was highly feasible concerning recruitment and the compliance was considered satisfactory. We found that the participants in both the water exercise and control group were highly physically active at follow-up. In the secondary outcomes, no significant differences were found between groups in the number of days on sick leave, in disability caused by low back pain, or in self-rated general health. Few women reported side effects, and only one woman withdrew from the trial, as she found the exercises uncomfortable to perform.

When interpreting the results of this trial, one must take into consideration that the proportion of healthy women with low back pain at 32 weeks (82%) was similar to other studies [11–14], but surprisingly our participants had a much lower intensity of pain (mean 2.01 vs 2.38 at follow-up) than other studies with low back pain [13,36,37]. Our participants were physically active to a large extent (89 and 85% respectively at 32 weeks of gestation) which has been shown to be associated with less pain in observational studies and might explain the low pain intensity among our participants [18]. This means that a reduction of 2, which is considered clinically significant on a numeric rating scale [33] would not have been feasible in our study population. We showed a mean difference of 0.38 on the primary outcome between the two groups. This is a small effect and we cannot based on our results conclude that the AquaMama program has a clinically significant effect on low back pain intensity. Despite the somewhat modest effect found we think that women may benefit from exercises in water. Thus more women in the water exercise group reported no low back pain at follow up 21% vs 14% respectively, the result was borderline significant (p = 0.07).

The increased mobility of the pelvic joints in pregnant women is considered one of the causes of low back pain [7] and specific muscle strengthening exercises are recommended as treatment for this condition [38]. In the present study, we aimed at strengthening the larger muscle groups, which may explain the effect found. Other studies have shown that stabilizing exercises have a positive effect on pain intensity in pregnant women with pelvic girdle pain and low back pain [9,37]. Furthermore, water buoyancy allows greater freedom of movement, making it easy to do multiple repetitions of exercises even in late pregnancy [39] and might explain why pregnant women benefit from this water exercise intervention [14,15]. Another explanation might be that anxiety has been shown to increase the pain intensity [1] and our participants experienced a sense of well-being with the water exercise [23].

Our findings of an unsupervised exercise water exercise are in line with two previous RCT [14,15] which investigated the effect of supervised exercises in water on low back pain and also found an effect on low back pain intensity. The studies did not report a mean difference, which made it difficult to compare them with ours, and they were considered of low methodological quality (no control group, no intention-to-treat analyses, randomised by date of birth). Three other RCTs [13,37,40] investigated the effect of a supervised land-based exercise intervention (stabilizing exercises, walking/exercises and water-gymnastics) and also showed a positive effect on pain intensity in pregnant women with low back pain. Two of the studies reported a mean difference of 1.7 and 2.9 respectively [37,40] but there were some methodological limitations; no description of randomization and statistical calculations in one [40] and no definition of low back pain and length of the intervention in the other [37]. The study by Kluge and colleagues [13] was small (n = 50) and only reported the difference from baseline to follow-up in the intervention group. One large (n = 386) RCT performed by Elden and colleagues [9] investigated the effect of stabilizing exercises performed both supervised and at home and found a mean difference of 9 on a VAS scale which is in line with our findings. Women were only included if they had been diagnosed with pelvic girdle pain.

We found that more women in the water exercise group stated no low back pain at follow-up, although this was only borderline statistically significant (p = 0.07). This is in line with a study by Eggen and colleagues, who also found a trend towards fewer women indicating low back pain after an exercise water exercise (OR 0.77, 95% CI = 0.50 to 1.19) [11]. Two other studies found a statistically significant difference in the number of women stating low back pain at follow-up [10,15], the study by Granath and colleagues used a different definition of low back pain which may explain the different results [15]. A large RCT (n = 855) with one weekly supervised exercise session did not find a difference in the number of women reporting low back pain (74 vs 75% p = 0.86) [12].

As opposed to other studies [12,14,15] we found no difference in the number of days spent on sick leave nor in the proportion of women being on sick leave due to low back pain between groups. Possible explanations for this result could be that the women spent a median of 4 days on sick leave (all causes) from 20–32 weeks of pregnancy, which is very few compared to other studies [10,12] and that our population was very healthy (highly educated, exercisers, non-smokers and non-obese).

### Limitations

Limitations of the trial are related to the generalizability, as the participants were very healthy, had higher education, low BMI, were non-smokers, lived with a partner and were physically active at baseline: they were therefore not representative of the general population. In this study, we excluded women suffering from pelvic girdle syndrome, which occurs in approx. 5% of all pregnant women, and the results are therefore not generalizable to these women. The reason for excluding women with a BMI > 29 kg/m^2^ was that another water exercise study was recruiting participants with BMI > 29 kg/m^2^ simultaneously with the present trial.

Neither investigators, nor participants were blinded to group allocation, as the nature of the water exercise made it difficult, this is considered a limitation. The exercise program used as part of the water exercise in this trial was publicly available, and as swimming and exercise in water tend to be the preferred type of exercise for pregnant women, this might have led to crossover: 17 women (6.6%) from the control group stated that they were engaged in water exercise at follow-up. The proportion of women who might have crossed over was small and we do not know if they followed the AquaMama program or another program, we therefore believe that this did not affect the result of this trial. At follow-up we found that a higher proportion of women performed resistance training, other exercise and brisk walking in the control group than in the intervention group. This could potentially have biased the result, since exercise in general is associated with lower risk of low back pain [18]. After excluding, women engaged in resistance training and other exercise the low back pain intensity score however remained similar indicating no bias by these types of exercise. Because of the high proportion of women engaged in brisk walking (n = 175) we were not able to perform robust analyses excluding these women. It is a weakness in randomized controlled trials with physical activity as intervention that physical exercise in general is recommended and therefore cannot be isolated to the intervention group. This makes it difficult to know if water exercise per se was the reason for the effect found. However, the largest difference between the groups was water exercise (p< 0.001) and we found it most likely that the modest but significant effect found was due to the water exercise intervention. The participants of this trial had very little disability due to low back pain (RMDQ) [28], with a median of 0 in both groups at baseline and 1 at follow-up on a scale from 0–23, which means that a significant reduction was not achievable. The RMDQ was developed for people with low back pain and it must therefore be taken into consideration whether this instrument was appropriate for a healthy population of pregnant women. The study may be considered to be overpowered, but this is due to deviations from two assumptions in our sample size estimate; first there was an unexpected low dropout rate, second the response rate at follow up was higher than expected, even though we stopped recruiting before initially planned. This larger study population may contribute to the statistically significant effect found, but is unlikely to affect the clinical effect of the intervention and therefore does not affect the conclusion of the study. The clinical implication of our trial is that healthy pregnant women can benefit from an unsupervised exercise program and it provides health professionals with an opportunity to recommend a type of exercise that has a documented positive effect on low back pain and is safe for pregnant women. Our exercise intervention is in accordance with the latest recommendations from The American Congress of Obstetricians and Gynecologists 2015 [41] who states that women with uncomplicated pregnancies should be encouraged to engage in moderate-intensity (Borg 13–14) exercise during pregnancy and that strengthening of abdominal and back muscles could minimize the risk of low back pain. Swimming is mentioned as a safe activity that can be initiated or continued during pregnancy [42].

This study was highly feasible, the compliance was considered good (74% participated in half or more of the exercises sessions) and only one participant withdrew from the water exercise group as she found the exercises uncomfortable to do, showing that not only does this water exercise comply with international recommendations, it also seems to appeal to pregnant women. Unsupervised exercise offers women a greater freedom to exercise when convenient and it therefore allows for more individual planning, which should be balanced with the benefits of supervised exercise.

In conclusion, we found that an unsupervised water exercise program results in a statistically significant lower intensity of low back pain, but the result was most likely not clinically significant. We found no effect on sick leave, disability due to low back pain or self-reported general health. Furthermore we found the program to be feasible and safe to do in a population of healthy pregnant women. For future studies, it is important to perform a cost-effectiveness analysis of the water exercise program and to investigate whether this water exercise has an effect on the general population, including pregnant women with higher pain scores and who are less physically active.

## Supporting information

S1 CONSORT checklist (PDF)

S1 Protocol (PDF)
