# Supplementary material for: The effects of an unsupervised water exercise program on low back pain and sick leave among healthy pregnant women – A randomised controlled trial
Source: PLoS One. 2017 Sep 6;12(9):e0182114. doi: 10.1371/journal.pone.0182114 (PMC5587299; doi:10.1371/journal.pone.0182114)
Supplement: S1 Protocol — (PDF) [file pone.0182114.s002.pdf]

Protocol
Title: Pregnancy and water exercise

Introduction

During pregnancy up to 50-75% of all pregnant women experience low back pain in the Scandinavian countries (1-4). It is problematic on both personal and socio-economic level, as low back pain reduces the possibilities to carry out daily activities (5, 6). Moreover, low back pain is associated with both increased risk of sick leave during pregnancy and low back pain after birth (7-11). A report from the Danish Ministry of Employment showed that almost 2/3 of all pregnant women were on sick leave beyond the last four weeks before the birth, where all pregnant women have the right to maternity leave (12).

Few and very different studies worldwide have investigated whether low back pain during pregnancy can be prevented or reduced (13-18). Many women reduce physical activity level during pregnancy and discontinue strenuous physical activities (19-21), as they find it unsafe or uncomfortable to perform during pregnancy (22; 23). Many women find it pleasant to perform activities such as swimming or water gymnastics and studies have shown that these activities increases temporarily during pregnancy (19; 23). On this basis, we want to investigate whether exercise activities in water have a positive effect on low back pain and sick leave during pregnancy.

Our hypothesis is that a part of the pain can be prevented or reduced by moderate exercise during pregnancy. The Danish Rheumatism Association has developed the AquaMama concept for pregnant women. AquaMama concept consists of six exercises designed specifically for pregnant and intended for unsupervised exercise in water. The tools necessary for performing the exercises are available in more than 130 public swimming pools in Denmark. Furthermore, many lifeguards are certified AquaCoaches and can supervise and advise the pregnant women while performing the exercises.

In this study we will therefore investigate whether water exercise with the AquaMama concept during pregnancy has an effect on the intensity of low back pain and the number of days of sick leave.

We expect that this study leads to new knowledge about whether the exercise in water can affect the pregnant woman's low back pain and sick leave in a positive way. The new knowledge can be used in antenatal care in Denmark. Furthermore, it is our hope that the new knowledge can be an inspiration in municipalities and workplaces.

Background

Low back pain is experienced in up to 50-75% of all pregnant women in the Scandinavian countries (1-4). The pain is costly, as it affect the pregnant woman's ability to perform daily activities (5; 6) and is associated with sick leave. Previous studies have shown that a high proportion of days spend on sick leave is related to pain in the musculoskeletal system, and that more than 25% of the days spend on sick leave during pregnancy can be attributed to low back pain (10; 11). Several studies have shown that low back pain during pregnancy is associated with low back pain for several years after birth (7; 8; 24). Few studies worldwide have investigated whether low back pain in pregnancy can be prevented or reduced (13-18).

The National board of Health recommends that healthy pregnant women should be moderately

physically active at least 30 minutes a day (25). Physical activity during pregnancy reduces the risk of many pregnancy and birth complications (25-29). However, the majority of women decrease their physical activity level during pregnancy compared to before pregnancy, and many stop strenuous exercise activities (19-21). Causes may be the growing body complications or insecurity. Activities such as swimming and water exercise is the type of activities which increases during pregnancy, and pregnant women feel comfortable performing it.(19; 23; 30; 31). Recent studies have shown that swimming during pregnancy was not associated with higher risks for pregnancy complications (32; 33). The National Board of Health recommends non-weight-bearing physical activity for women with back or pelvic pain (25).

While the explanation for low back pain during pregnancy can be attributed to some muscle groups are more relax while others become more tense as a result of a change partly to the body's center of gravity and a woman's weight (34), it is obvious that physical activities, especially physical activities in water, which gives the pregnant woman a greater ability to move (23) may reduce the intensity of low back pain. It is important to distinguish between low back pain, and pelvic girdle syndrome, as the prognosis and the treatment is different (39). Pelvic girdle syndrome occurs in approximately 5% of pregnant women in Denmark, usually occurs around week 20 and gradually worsened during pregnancy (40). Pelvic girdle syndrome is defined as pain localized to the 3 sacroiliac joint. The prognosis for pelvic girdle syndrome is worse than for low back pain, as about 20% is still not free of pain two years after birth (41). Pelvic girdle syndrome is diagnosed by a physiotherapist, performing an examination of joints and muscles of the pelvic region, to ensure that the pain is joint- related and not muscular.

The evidence on the prevention and reduction of low back pain during pregnancy is very limited. Two Swedish studies found positive effects of exercise in water during pregnancy on low back pain, pelvic pain and sick leave (14; 35). A weakness in one study was that there were included women with both low back pain and pelvic pain (14). As the two syndromes have different etiology, treatment and prognosis, it may be misleading to assess the effect as a whole. Neither of the two studies described the intervention in details, which is necessary if the studies, is to be reproduced. In both studies the training took place at specific time, during daytime, and participants indicated having difficulty to follow a specific team. In one study the water training (intervention) took place in hot water (35), such a study is not possible to appliqué in Denmark as hot pools only exist in a limited number in Denmark.

We believe that the Aqua Mama program is a suitable for an intervention study in Denmark. Unlike the Swedish studies, it is a form of intervention that is individual and can be adapted to work and family and Aqua Mama can take place in a normal public swimming pool. It is important to test a program that is available (geographic / economic) for all pregnant women. The standardized Aqua Mama exercise series is developed by professionals, so the woman has a sense of security by conducting the exercises. A standardized training program also increases the generalizability.

Hypothesis and objectives

The hypothesis is that water exercise with the Aqua Mama program twice a week of 45 minutes duration for 12 weeks during pregnancy will reduce the intensity of the pregnant woman's lower back pain and reduce the number of days of sick leave.

The project objective is to investigate the effect of water exercise with Aqua Mama program during pregnancy on the intensity of low back pain and duration of sick leave.

Method

Design

The study is built up as a randomised controlled study of pregnant women with unsupervised exercise with the Aqua Mama program as intervention. The women are randomized in a ratio of 1: 1 for the two groups (intervention versus control). Randomisation is via a specially designed computer system. Randomization is pre-arranged with the Centre for Public Health and Quality (CFK), Central Jutland, where randomization will be stored.

Participants'

Healthy pregnant women with a normal pregnancy with a single fetus.
Inclusion Recorders Does the:

1) Women ≥18 years
2) Informed consent
3) Gestational age 14-17 weeks
4) and understand Danish
5) Women with BMI ≤ 29 kg/m^2
6) Pregnant women with a chronic disease before pregnancy is offered first participation by prior arrangement with the obstetrician at the hospital
7) Can swim 50 meters

Exclusion criteria:
1) <18 years
2) Multiple pregnancies
3) Abuse Problems
4) Pelvic girdle syndrome in previous pregnancies (diagnosed by physical therapist or doctor)
5) Conditions or chronic diseases where physical activity is contraindicated

Intervention

Participants are offered:
• an introduction session with a midwife, physiotherapist and Aqua Coach on water exercise with the Aqua Mama program
• There will be introductory sessions each month. There will be a midwife or physiotherapist present at all sessions
• Women are encouraged to exercise with Aqua Mama program twice per week by 45 minutes duration of 12 weeks.

Content and dosage of self-training: The pregnant participants perform six exercises like circuit training in two series. In each series performed Mama Surf 2 x 20-30 times, Mama Pendulum 2 x15-20 times, Mama Boxing 2x15-20 times MamaBiceps 2x15-20 times, Mama Lift 2x10-12 times and Mama Jogging 2x2 min and exercises done at a steady pace Furthermore the participants will swim 4 laps before and after the exercise program (200 m total). The program has been tested by a physiotherapist.
The exercises can be found at:
http://www.gigtforeningen.dk/aquapunkt/aquamama+%c3%b8velser.

• Each month there will be a brush-up session for participants, at the session there will be a Aquacoach, a midwife and physiotherapist present in the swimming pool.
• Providing a free voucher with 12 tickets to the swimming pool.

In order to support the compliance and adherence, the following is carried out (23, 44-45):
1) The participants assembled and introduced to each other at the introductionssession
2) Participants receive weekly motivational SMS and e-mails from project midwife
3) Participants can see graphs with each other's training sessions on the website. This is optional and for those women who want a little competition.
4) There will be created a Facebook group where participants can give each other ideas and share experiences with the exercises and find training partners
5) Via e-mail or SMS participants send messages to project the midwife about the number of training times per week as well as experiences with training

Indications for treatment stops
-PPROM (Rupture of membranes before the 37th week of pregnancy)
-Symptomatic placenta praevia
-Pelvic girdle syndrome, diagnosed by a physiotherapist from international guidelines
-Vaginal Bleeding
-Threatening premature birth
-Preeclampsia

Control group
Will receive general guidance and advice on exercise as part of standard prenatal care. The control group will receive two free tickets for baby swimming, when the final questionnaire has been filled out at follow up.

Outcome variables / endpoints
Outcome variables / endpoints measured in the 32nd week of pregnancy. Primary end-point is the intensity of low back pain.
Secondary end-points are: Sick leave (yes / no), number of days and the cause of sick leave, physical disability and general health experienced in the 32nd week of pregnancy.

Questionnaire (Measured at baseline and at 32 weeks of gestation)
The participants will be presented with a drawing of a women where the definition of low back pain is illustrated.

Low back pain rating Scale
Is designed to detect changes in the level of pain in the low back (36). It consist of a 11-point box scales. 3 scales measuring pain "just now", "the worst back pain" within the last 14 days and "average" back pain within the last 14 days. Highest figures represent the worst pain (36).

Sick leave
The number of days of sick leave and cause / causes of absenteeism are stated

Roland Morris Disability Questionnaire (RMDQ)

Roland Morris Disability Questionnaire (RMDQ) is a questionnaire investigating functional capacity in patients with low back pain. The scale measures the impaired function due to back problems. It consists of 23 questions that are answered with yes / no. Is given the maximum 23 points, where the highest score indicates worst disability (37).

General health

Health-related quality of life is measured EQ-5D and thermometer Points are given from 0 to 100 (38).

General statistical considerations

The primary data analysis will be performed based on the intention-to-treat principle.
Baseline data (parity, previous low back pain) for the two groups will by compared: For quantitative data a t-test will be applied if the data are sufficiently normal, otherwise the Kruskal-Wallis test will be applied. Qualitative data will be compared by the Fisher or chi-square test, the latter being applied if the condition of the expected values being larger than 5 is fulfilled.
A t-test will be applied to compare the mean of the primary outcome in the two groups. Similarly secondary outcomes will be compared using t-tests. For the secondary outcomes, the p-values will be adjusted for multiple testing using the Benjamini-Hochberg correction method (ref). If the data are not sufficiently normally distributed, a transformation will be applied if possible.
To explore the impact of parity and previous low back pain score on the outcomes, additional linear regression analyses of the outcomes will be performed. In case of influential observations (probably only relevant for number of days on sick leave), robust regression techniques will be applied. Per- protocol analysis will be performed of the pregnant women who perform ≥ 75% of all training sessions. The significance level will set at 0.05.

Sample size assessment

Sample size calculation was based on the following conditions:

2 points reduction on a numerical pain scale of 0-10, for the patient experiencing a reduction in pain as clinically important.

- The Average highest pain at baseline is 5.8 and SD = 3 (3; 35). –A total of 70% of all pregnant women who experience low back pain.

- We assume that the effect of the intervention is 2.0 points if all participants complete the full program.

- It is estimated that 50% of participants follow the program for at least 75% of the sessions (high participation, 19-24 sessions) while 35% follow 50-75% (moderate participation, 12 - 18 sessions) and 15% follow less than half of the program (low participation). We estimate that the effect of high participation in the training course is a reduction of 2 points, the effect of moderate participation in the training course is a reduction of 1 point, while low participation does not result in a reduction. This gives an overall reduction in the intervention group of 1.35 points (0.50 * 2 + 0.35 * 1 + 0.15 * 0 = 1.35). A difference in pain level of 1.35 (SD = 3.0) will be available with a power of 90% and a significance level of 5% (two-sided) with 150 participants in each group.

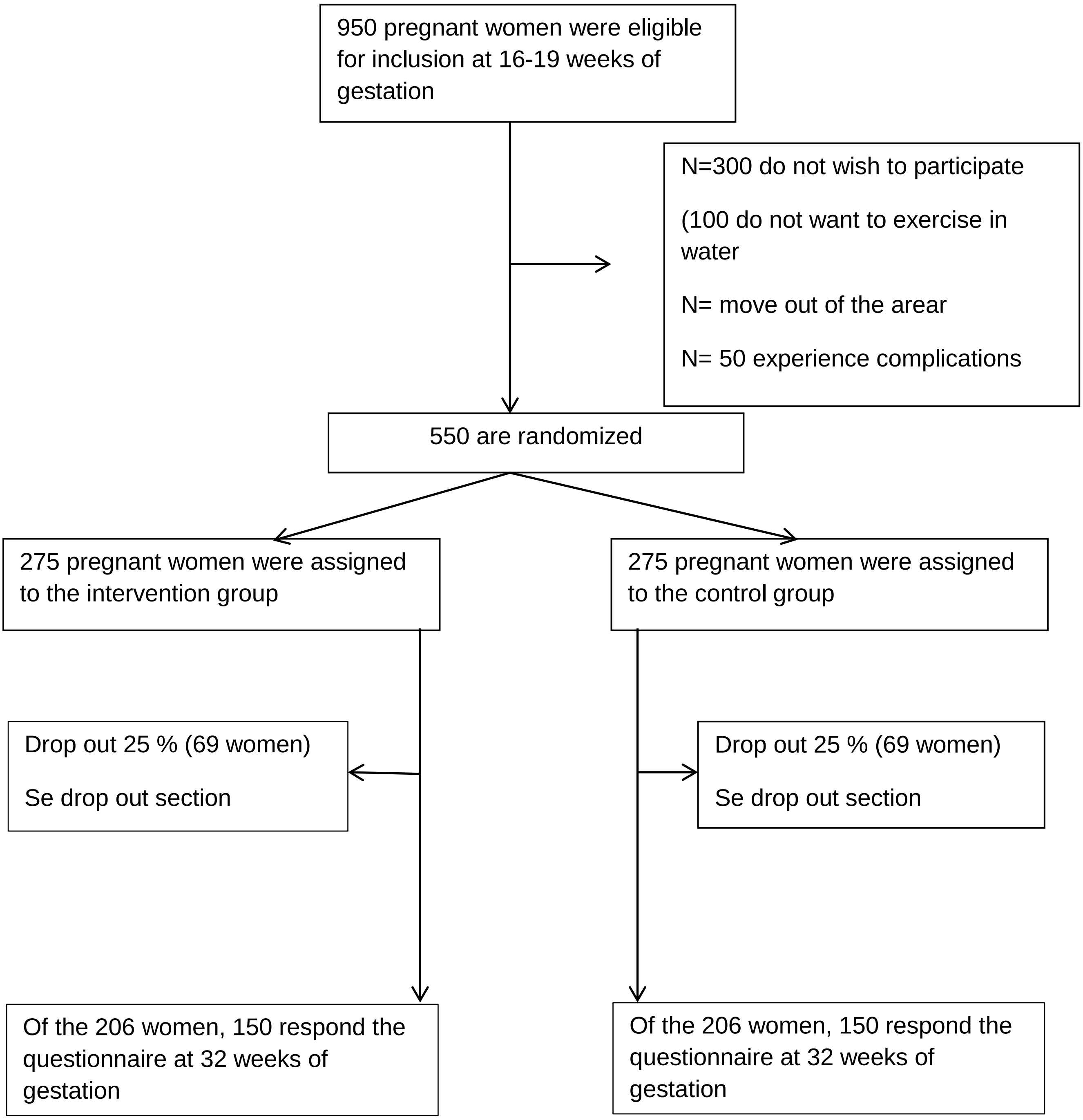
950 pregnant women were eligible for inclusion at 16-19 weeks of gestation
N=300 do not wish to participate
(100 do not want to exercise in water
N= move out of the arear
N= 50 experience complications
550 are randomized
275 pregnant women were assigned to the intervention group
275 pregnant women were assigned to the control group
Drop out 25 % (69 women)
Se drop out section
Drop out 25 % (69 women)
Se drop out section
Of the 206 women, 150 respond the questionnaire at 32 weeks of gestation
Of the 206 women, 150 respond the questionnaire at 32 weeks of gestation

Strategy in case of missing outcomes

For outcomes with less than 5 percent missing data, complete case analyses will be reported. If the percentage of missing data exceeds 5, a sensitivity analysis based on worst-best and best-worst imputation will be applied (in the experimental arm imputing the worst / best outcomes observed in the control arm to the missing outcomes and vice versa for the control arm). In case these analyses give rise to the same conclusion as the complete case analyses, the latter will be reported. Otherwise data will be assumed Missing At Random (MAR) and analyses will be performed using Maximum-Likelihood or Inverse Probability Weighting.

Primary outcome	Description	Handling of partly missing outcomes
Low back pain	Three numeric 11 point box scales (pain now, worst pain in the past two weeks, average pain in the past two weeks), 0 indicating no pain to 10 indicating worst pain imaginable.	
Secondary outcome		
Days spend on sick leave	Number of days spend on leak leave,<br>1. Due to pregnancy-related complications.<br>2. Sickness or conditions with no relation to the pregnancy.<br>3. Due to the work environment.	
Roland Morris Disability Questionnaire	A 23 item disease specific instrument developed to asses physical disability caused by low back pain.<br>Conversion of yes/no answers to a 0-100 scale. The answers is converted to percentages	When two answers ore more are missing the raw sum score will also be converted to percentages.
EQ-5D + termometer	General health status covering the five dimensions; mobility, self-care, usual activities, pain/discomfort and	

	anxiety/depression. The five dimensions are each assessed by a single answer on a three-point ordinal scale (no problems, some problems, extreme problems).<br><br>Self-rated quatity of life reated on a o-100 point scale	

Recruitment of participants.
Participants in the study will be healthy pregnant women who follow the antenatal care at Rigshospitalet, Copenhagen Denmark. As part of the antenatal care pregnant women fill out a standard questionnaire regarding health, previously pregnancies and lifestyle for the use in the antenatal care. Based on this information, participants will be selected for the study. The first contact to the participant is made by letter, containing written information about the trial. After one week the women will be contacted by a research midwife and invited to a face-to face meeting, if the woman is interested in hearing more about the trial.
At the meeting. which is held at Rigshospitalet in 16-17 weeks, the woman will receive oral information about the trial by a the research midwife. The conversation takes place in an enclosed space and the woman will get up to 30 minutes of second to decide whether she will participate in the trial. This reflection is considered acceptable when the woman already has had the opportunity to consider its participation. During the conversation in 16-17 weeks of pregnancy will obtain consent.

Dropout
Women with Pelvic girdle syndrome are excluded from the trial. If Pelvic girdle syndrome is suspected, the women will have a physical examination by research midwife, trained by physiotherapist PhD Hanne Albert, to exclude women with Pelvic girdle syndrome. If in doubt, the woman will be examined by a physiotherapist, committed to the project. The dropout rate in the intervention group is expected to be modest, with only 5% of all pregnant women diagnosed with Pelvic girdle syndrome. When pain occurs early in pregnancy around pregnancy week 20, and therefore will be diagnosed at baseline, there will be only 1-2% will develop pain after inclusion in the trail. Furthermore a dropout rate of 23% of participants due to other complications: 10% of pre-term birth or picking pangs 5% that gets hypertensive complications, and 13% to other complications (vomiting, placenta praevia, stress, bleeding ect.). A total dropout rate of 25% due to pregnancy-related complications is expected.

Data Collection
All information regarding participants is protected by law concerning the processing of personal data and Law about patients' legal position.
Background Data: Prior to randomization collected information about the woman's socio-demographic characteristics, height and weight, lifestyle factors (smoking, exercise, alcohol.),

Abuse and medical, gynecological and obstetric history, incl. fertility treatment, previous preterm birth and previous pelvic instability diagnosed by a physical therapist or doctor. This information is collected as part of the general systematic collection of medical records, which were held in Obstetric Clinic at Rigshospitalet.
Data on the current pregnancy and delivery: For all participants gathered information about complications in pregnancy and during labor, gestational age at birth, the children's weight and length at birth, and children's condition immediately after birth. These data are collected by chart review after birth. Information on low back pain and sick leave: Information collected in a questionnaire handed out at baseline in 16-17 weeks of pregnancy and sent to the participating women at follow up in the 32nd week of pregnancy.

Time schedule

01.11.2012 - 01.04. 2013: Implementation and preparation of studies and pilot study with 40 participants. Interview of 10 participants
01.11.2013 to 01.02. 2015: Inclusion of 550 participants, representing 10 -11 pregnant per week,
01.3. 2015 to 01.09.2015: Collection of remaining questionnaires from 32.graviditetsuge and data relating to pregnancy and childbirth process. Data analysis, writing articles and sending scientific papers to the journal.
Interviews with participants in the pilot phase
10 women will be interviewed from the pilot project on intervention component based on a semi-structured interview guide The themes are the assistance elements and motives to participate in the project pregnancy and water training. The interviews will be conducted individually with a duration of 20-45 minutes and take place by telephone. Participants to be interviewed will be selected at random. The interviews will be transcribed and analyzed by content analysis.

Ethical aspects

Review of data processing entitled "Pregnancy and water exercise" is authorized and declared the Capital Region's umbrella review of health research with j.nr .: 200758-0015. The local j.nr. the unit is: 30-0828. The participants give informed consent under current law. Act on processing of personal data will be respected.
It is considered ethically acceptable to offer water exercise with the Aqua Mama concept to the participants in the intervention group, as studies on water exercise has shown no adverse effects on pregnant women nor the fetuses. It is also expected that participation in the study will be useful to the subjects and for women in the future. Women in the control group will receive general guidance and advice on exercise in antenatal care

Collaborators

The project collaborates applicant physiotherapist Ph.D. Hanne Albert, who is an experienced researcher in pregnancy and low back pain and the use of measuring instruments for the measurement of back pain and rygfunktionsniveau. The other three partners in the project are Professor, MD. Peter Damm, Professor, MD. Ann Tabor and Clinic, Ph.D. Morten Hedegaard, Obstetrics Clinic, Rigshospitalet. All three have great scientific experience and has completed several epidemiological and clinical randomized controlled studies.
Team Bade in Copenhagen, who manage the public swimming pools in Copenhagen have agreed on a monthly information and introductory sessions with Aqua Mama in Øbro Hallen.

What new knowledge expected the project to give

The project is expected to provide new knowledge of the effect of water exercise in healthy pregnant women on low back pain and sick leave. It will also provide knowledge of compliance of unsupervised exercise. This knowledge will be used by midwifes and doctors in antenatal care.

Publication of test results

The project results are expected to be disseminated in the peer-reviewed American obstetric journal "Obstetric and Gynaecology". After publishing the survey is expected to be discussed in trade journals for doctors, physiotherapists and midwives. There will be shipped items to patient organizations and consumer magazines. In addition, there will be a contact magazine in Danish municipalities. The results will be published regardless of the outcome of the trial. The results will be published as soon as possible.

Economic conditions

The midwife Hanne Kristine Hegaard, Rigshospitalet, who initiated the study. The study is funded by TrygFonden, The Danish Rheumatism Association, the Danish Midwives Association's research and development, Augustinus Foundation and The Lundbeck Foundation.

Reference List

(1 Bjorklund K, Bergstrom S. Is pelvic pain in pregnancy a welfare complaint? Acta Obstet Gynecol Scand 2000 Jan;79(1):24-30.

(2) Kristiansson P, Svardsudd K, von SB. Back pain during pregnancy: a prospective study. Spine (Phila Pa 1976) 1996 Mar 15;21(6):702-9.

(3) Mogren IM, Pohjanen AI. Low back pain and pelvic pain during pregnancy: prevalence and risk factors. Spine (Phila Pa 1976) 2005 Apr 15;30(8):983-91.

(4) Ostgaard HC, Andersson GB, Karlsson K. Prevalence of back pain in pregnancy. Spine (Phila Pa 1976) 1991 May;16(5):549-52.

(5) Mogren I. Perceived health, sick leave, psychosocial situation, and sexual life in women with low-back pain and pelvic pain during pregnancy. Acta Obstet Gynecol Scand 2006;85(6):647-56.

(6) Olsson C, Nilsson-Wikmar L. Health-related quality of life and physical ability among pregnant women with and without back pain in late pregnancy. Acta Obstet Gynecol Scand 2004 Apr;83(4):351-7.

(7) Gutke A, Ostgaard HC, Oberg B. Predicting persistent pregnancy-related low back pain. Spine (Phila Pa 1976) 2008 May 20;33(12):E386-E393.

(8) Noren L, Ostgaard S, Johansson G, Ostgaard HC. Lumbar back and posterior pelvic pain during pregnancy: a 3-year follow-up. Eur Spine J 2002 Jun;11(3):267-71.

(9) Stapleton DB, MacLennan AH, Kristiansson P. The prevalence of recalled low back pain during and after pregnancy: a South Australian population survey. Aust N Z J Obstet Gynaecol 2002 Nov;42(5):482-5.

(10) Sydsjo A, Sydsjo G, Wijma B. Increase in sick leave rates caused by back pain among pregnant Swedish women after amelioration of social benefits. A paradox. Spine (Phila Pa 1976) 1998 Sep 15;23(18):1986-90.

(11) Sydsjo A, Sydsjo G, Alexanderson K. Influence of pregnancy-related diagnoses on sick-leave data in women aged 16-44. J Womens Health Gend Based Med 2001 Sep;10(7):707-14.

(12) Analyse af graviditetsbetinget fravær, Beskæftigelses Ministeriet 2010. 2011.
Ref Type: Generic

(13) Garshasbi A, Faghih ZS. The effect of exercise on the intensity of low back pain in pregnant women. Int J Gynaecol Obstet 2005 Mar;88(3):271-5.

(14) Granath AB, Hellgren MS, Gunnarsson RK. Water aerobics reduces sick leave due to low back pain during pregnancy. J Obstet Gynecol Neonatal Nurs 2006 Jul;35(4):465-71.

(15) Kluge J, Hall D, Louw Q, Theron G, Grove D. Specific exercises to treat pregnancy-related low back pain in a South African population. Int J Gynaecol Obstet 2011 Jun;113(3):187-91.

(16) Shim MJ, Lee YS, Oh HE, Kim JS. Effects of a back-pain-reducing program during pregnancy for Korean women: a non-equivalent control-group pretest-posttest study. Int J Nurs Stud 2007 Jan;44(1):19-28.

(17) Ekdahl L, Petersson K. Acupuncture treatment of pregnant women with low back and pelvic pain--an intervention study. Scand J Caring Sci 2010 Mar;24(1):175-82.

(18) Pennick VE, Young G. Interventions for preventing and treating pelvic and back pain in pregnancy. Cochrane Database Syst Rev 2007;(2):CD001139.

(19) Haakstad LA, Voldner N, Henriksen T, Bo K. Physical activity level and weight gain in a cohort of pregnant Norwegian women. Acta Obstet Gynecol Scand 2007;86(5):559-64.

(20) Hegaard HK, Damm P, Hedegaard M, Henriksen TB, Ottesen B, Dykes AK, et al. Sports and Leisure Time Physical Activity During Pregnancy in Nulliparous Women. Matern Child Health J 2010 Aug 1.

(21) Owe KM, Nystad W, Bo K. Correlates of regular exercise during pregnancy: the Norwegian Mother and Child Cohort Study. Scand J Med Sci Sports 2008 Jun 17.

(22) Duncombe D, Wertheim EH, Skouteris H, Paxton SJ, Kelly L. Factors related to exercise over the course of pregnancy including women's beliefs about the safety of exercise during pregnancy. Midwifery 2007 Dec 4.

(23) Hegaard HK, Kjaergaard H, Damm PP, Petersson K, Dykes AK. Experiences of physical activity during pregnancy in Danish nulliparous women with a physically active life before pregnancy. A qualitative study. BMC Pregnancy Childbirth 2010;10:33.

(24) Stapleton DB, MacLennan AH, Kristiansson P. The prevalence of recalled low back pain during and after pregnancy: a South Australian population survey. Aust N Z J Obstet Gynaecol 2002 Nov;42(5):482-5.

(25) www.sst.dk. 2009.
Ref Type: Internet Communication

(26) Hegaard HK, Pedersen BK, Nielsen BB, Damm P. Leisure time physical activity during pregnancy and impact on gestational diabetes mellitus, pre-eclampsia, preterm delivery and birth weight: a review. Acta Obstet Gynecol Scand 2007;86(11):1290-6.

(27) Hegaard HK, Hedegaard M, Damm P, Ottesen B, Petersson K, Henriksen TB. Leisure time physical activity is associated with a reduced risk of preterm delivery. Am J Obstet Gynecol 2008 Feb;198(2):180-5.

(28) Juhl M, Andersen PK, Olsen J, Madsen M, Jorgensen T, Nohr EA, et al. Physical exercise during pregnancy and the risk of preterm birth: a study within the Danish National Birth Cohort. Am J Epidemiol 2008 Apr 1;167(7):859-66.

(29) Owe KM, Nystad W, Bo K. Association between regular exercise and excessive newborn birth weight. Obstet Gynecol 2009 Oct;114(4):770-6.

(30) Evenson KR, Savitz DA, Huston SL. Leisure-time physical activity among pregnant women in the US. Paediatr Perinat Epidemiol 2004 Nov;18(6):400-7.

(31) Hegaard HK, Petersson K, Hedegaard M, Ottesen B, Dykes AK, Henriksen TB, et al. Sports and leisure-time physical activity in pregnancy and birth weight: a population-based study. Scand J Med Sci Sports 2009 Apr 14.

(32) Juhl M, Kogevinas M, Andersen PK, Andersen AM, Olsen J. Is swimming during pregnancy a safe exercise? Epidemiology 2010 Mar;21(2):253-8.

(33) Nieuwenhuijsen MJ, Northstone K, Golding J. Swimming and birth weight. Epidemiology 2002 Nov;13(6):725-8.

(34) www.gigtforeningen.dk. 2009.
Ref Type: Internet Communication

(35) Kihlstrand M, Stenman B, Nilsson S, Axelsson O. Water-gymnastics reduced the intensity of back/low back pain in pregnant women. Acta Obstet Gynecol Scand 1999 Mar;78(3):180-5.

(36) Manniche C, Asmussen K, Lauritsen B, Vinterberg H, Kreiner S, Jordan A. Low Back Pain Rating scale: validation of a tool for assessment of low back pain. Pain 1994 Jun;57(3):317-26.

(37) Albert HB, Jensen AM, Dahl D, Rasmussen MN. [Criteria validation of the Roland Morris questionnaire. A Danish translation of the international scale for the assessment of functional level in patients with low back pain and sciatica]. Ugeskr Laeger 2003 Apr 28;165(18):1875-80.

(38) Pedersen K W-JKBRGC. Værdisætning af sundhed. 256. 2011. University of Southern Denmark Publishing, 2003. Ref Type: Serial (Book,Monograph)

(39) Vleeming A, Albert HB, Ostgaard HC, Sturesson B, Stuge B. European guidelines for the diagnosis and treatment of pelvic girdle pain.Eur Spine J. 2008 Jun;17(6):794-819.

(40) Albert HB, Godskesen M, Westergaard JG. Incidence of four syndromes of pregnancy-related pelvic joint pain.Spine (Phila Pa 1976). 2002 Dec 15;27(24):

(41) Albert H, Godskesen M, Westergaard J. Prognosis in four syndromes of pregnancy-related pelvic pain.Acta Obstet Gynecol Scand. 2001 Jun;80(6):505-10.

(42) Ostelo RW, Deyo RA, Stratford P, et al. Interpreting change scores for pain and functional status in low back pain towards international consensus regarding minimal important change. Spine 2008;33:90-4

(43) Haakstad LA, Bø K. Exercise in pregnant women and birth weight: a randomized controlled trial. BMC Pregnancy Childbirth. 2011 Sep 30;11:66.
